# eHealth for Patient Engagement: A Systematic Review

**DOI:** 10.3389/fpsyg.2015.02013

**Published:** 2016-01-08

**Authors:** Serena Barello, Stefano Triberti, Guendalina Graffigna, Chiara Libreri, Silvia Serino, Judith Hibbard, Giuseppe Riva

**Affiliations:** ^1^Department of Psychology, Università Cattolica del Sacro CuoreMilan, Italy; ^2^Applied Technology for Neuro-Psychology Lab, Istituto Auxologico ItalianoMilan, Italy; ^3^Department of Planning, Public Policy, and Management, University of OregonEugene, OR, USA

**Keywords:** eHealth, patient engagement, patient experience, patient activation, systematic review

## Abstract

eHealth interventions are recognized to have a tremendous potential to promote patient engagement. To date, the majority of studies examine the efficacy of eHealth in enhancing clinical outcomes without focusing on patient engagement in its specificity. This paper aimed at reviewing findings from the literature about the use of eHealth in engaging patients in their own care process. We undertook a comprehensive literature search within the peer-reviewed international literature. Eleven studies met the inclusion criteria. eHealth interventions reviewed were mainly devoted to foster only partial dimensions of patient engagement (i.e., alternatively cognitive, emotional or behavioral domains related to healthcare management), thus failing to consider the complexity of such an experience. This also led to a great heterogeneity of technologies, assessed variables and achieved outcomes. This systematic review underlines the need for a more holistic view of patient needs to actually engage them in eHealth interventions and obtaining positive outcomes. In this sense, patient engagement constitute a new frontiers for healthcare models where eHealth could maximize its potentialities.

## Introduction

Reducing risks and improving patient health outcomes are requirements currently faced by healthcare systems all over the world (Epstein et al., 2010; Gruman et al., 2010; Graffigna et al., 2013a). Furthermore, cuts in health funds and competition for budgets require enhanced efficacy and efficiency of healthcare services provision (Elf et al., 2015). Engaging patients in the responsible management of their health is widely acknowledged as a way to answer those challenges. Indeed, patients who are active and effective managers of their healthcare are demonstrated to obtain more positive clinical outcomes than patients who are disengaged and passive (Hibbard et al., 2007; Frosch and Elwyn, 2011; Greene and Hibbard, 2012; Barello et al., 2015a,b). Moreover, there is increasing agreement that patient engagement is a crucial factor for improving quality of care and increasing patient safety (Schwappach, 2010).

In order to fully understand patient engagement, and also to introduce some terms of this review, it is useful to differentiate this concept from others which literature has traditionally used to describe the underpinnings of the process of patients achieving an active role in their own healthcare.

Indeed, studies aimed at discussing the active role of patients have used—often interchangeably—different terms (i.e., patient engagement, patient activation, patient involvement, patient participation, patient adherence/compliance, and patient empowerment) when referring to this concept (Barello et al., 2014). Although, all these terms refer to the purpose of making patients more protagonists of their healthcare arena, research on this topic (Menichetti et al., 2014) suggest that each term is connoted by a peculiar meaning concerning the role that patients enact when called to relate with their own healthcare. For instance, “patient adherence” and “patient compliance” (Kyngäs et al., 2000; Vermeire et al., 2001) are interconnected and overlapping concepts, both focusing mainly on the behavioral components of the patients' care experience; “patient participation” and “patient involvement” (Guadagnoli and Ward, 1998; Wellard et al., 2003) mainly refer to a proficuos relational patient-doctor exchange which allows a shared treatment decision making; finally, “patient empowerment” (Aujoulat et al., 2007)—a concept which is deeply psychological in its nature—describes the patients' subjective sense of control over their own disease and treatment management and the feeling of being directly responsible for their own health outcomes. Within this field, the healthcare debate is currently focusing on the concept of “patient engagement”—called also “patient activation” (Hibbard et al., 2004; Gruman et al., 2010; Carman et al., 2013; Graffigna et al., 2013a, 2014a, 2015a). Mutated from the marketing literature (Gambetti and Graffigna, 2010; Hardyman et al., 2015), this concept is oriented by a consumer health perspective that considers patients as subjects involved in a specific socio-cultural context. For these reasons, patient engagement is different from the terms described above, as well as it refers to the different aspects (not only subjective, but also contextual, relational and organizational) that may foster or hinder the patients' ability to truly become positioned at the center of their own care. In other words, the concept of patient engagement offers a broader and better systemic conceptualization of the patients' role when interacting with their own healthcare (Menichetti et al., 2014; Hardyman et al., 2015). It is an “umbrella” term that qualifies the systemic relationship that occurs between the “supply” and the “demand” of healthcare—at different levels and in different contexts (Graffigna and Barello, 2015).

Carman et al. (2013) define patient engagement as “a set of behaviors by patients, family members, and health professionals and a set of organizational policies and procedures that foster both the inclusion of patients and family members as active members of the health care team and collaborative partnerships with providers and provider organizations with the desired goals of patient and family engagement include improving the quality and safety of health care.” This definition considers engagement as a systemic concept, which is the outcome of patient's actions carried out at different levels of complexity (i.e., individual, relational, organizational, and health policy).

Other scholars (Hibbard et al., 2004) define patient engagement in terms of level of “activation,” by connoting an engaged patient as “an active agent in the management of his/her own health.”

Similarly, Gruman's patient engagement behavioral framework (2010) has the value of ackowledging the different components of the patient engagement experience and defines it as the actions individuals may enact to participate knowledgeably and actively in their owb healthcare to realize its full benefit.

The dynamic nature of patient engagementis is described by Graffigna et al. (2013a; 2015a), who define this phenomenon as a “multi-dimensional psychosocial process resulting from the conjoint cognitive, emotional, and behavioral enactment of individuals toward their health condition and management.” These authors parlticularly underlines the role of the emotional elaboration—next to the behavioral and cognitive activation—as a crucial component in the process of patient becoming fully engaged in his/her own healthcare (Graffigna et al., 2013b).

According to all of these definitions, patient engagement is a complex and multi-faceted experience which cannot be reduced to the mere consideration of the patient's ability to adhere to medical prescriptions. Precisely, patient engagement is characterized by:

- *A Behavioral dimension* (What the patient *does):* connected to all the activities the patient acts out to face the disease and the treatments;
- *A Cognitive dimension* (What the patient *thinks* and *knows*): connected to what the patient knows, understands and how he/she makes sense of the disease, its treatments, its possible developments, its monitoring;
- *An Emotional dimension* (What the patient *feels*): connected to the psychological and emotional reactions the patients experience when adjusting to (and elaborating) the onset of the disease and new life condition linked to it.

Basing on this broader view of patient engagement, the variables involved in this process are heterogeneous and at different levels of the patient subjective experience. On the one hand, the experimental analysis of patient engagement can be sometimes reduced to some of its dimensions. On the other hand, it represents a risk to ignore some features of patient engagement, resulting in not being able to assess its complex dynamics when delivering and monitoring the outcomes of healthcare interventions devoted to this aim.

## The role of ehealth

**Figure 1:**
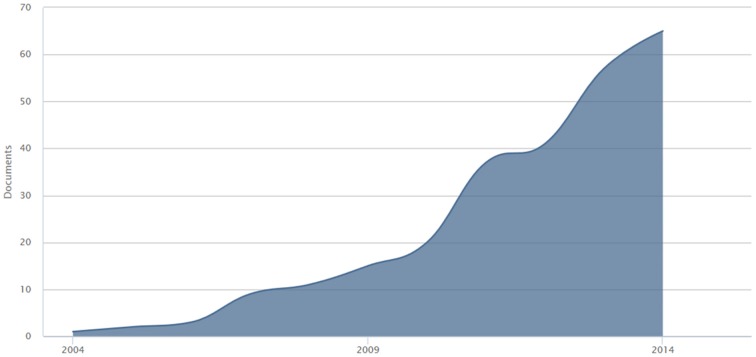
**Publication trend of eHealth for patient engagement studies across the last 13 years**.

In the field of healthcare interventions the new technologies for health (eHealth) are recognized to have tremendous potential for fostering patient engagement. These tools allow to develop integrated, sustainable and patient-centered services and promote effective exchanges among the actors involved in the care process (Eysenbach, 2001). This growing trend in the use of eHealth is clearly shown by the increasing number of published research on this topic in the last 13 years (see Figure 1).

eHealth is a broad term that encompasses a broad range of phenomena, conceptions and instruments. Up to now, a wide variety of definitions of this term are available in the literature (Eysenbach, 2001; Pagliari et al., 2005; Gorini et al., 2008; Graffigna et al., 2013a,b, 2014b,c; Cunningham et al., 2014; Gaddi and Capello, 2014), most of them highlighting the importance of Internet-related technologies to support, enable, promote and enhance health and augment the efficacy and efficiency of the process of healthcare. The most cited definition was found to be the one by Eysenbach (2001), which is significantly two-fold: on the one hand, he explains how the delivery of information to patients and stakeholders could be enriched by the intersection of medical informatics and public health business. On the other hand, he points out that not only a technical development is involved in the emerging of eHealth, but also a new state-of-mind marked by a global-thinking attitude and by the intention to improve health care locally, regionally and worldwide. To date, the majority of studies were designed to examine the efficacy of eHealth interventions in enhancing clinical outcomes both physical (Van den Berg et al., 2006; Norman et al., 2007) and psychological (Eland-de Kok et al., 2011). However, less attention has been devoted to the understanding of the impact of eHealth on patient engagement in their healthcare.

eHealth is addressed by the current literature as a valuable framework that favors the connection between the different systems and actors involved in the health management process, this way promoting patient engagement (Eysenbach, 2001; Ahern et al., 2008). However, in line with other scholars (Coulter and Ellins, 2007; Ricciardi et al., 2013), we emphasize the infancy of the literature debate on the relationship between eHealth implementations and patient engagement, and the lack of shared guidelines for orienting interventions in not only improving clinical effectiveness but also in making the patients' care experience positive, sustainable and oriented to achieve stable well-being.

Thus, the purpose of the current review is to detect, categorize and synthesize findings from the literature about the application of eHealth in engaging people in their own care process. The best practices, potential challenges and opportunities for the ability of eHealth interventions to foster patient engagement in existing healthcare systems are discussed below.

Indeed, the authors of this study are oriented by a complex and multi-componential vision of patient engagement which describes this phenomenon as featured by emotional, cognitive and behavioral aspects (see the Introduction section). For this reason, we searched for studies whose authors explicitely declared to focus on eHealth for patient engagement/activation.

This paper attempts to answer the following research questions:

- Which patient engagement outcomes are considered when describing the effects of eHealth interventions (i.e., variables)? How patient engagement outcomes are assessed in eHealth interventions (i.e., measures and methododology)?
- What dimensions (behavioral, cognitive, emotional) of the patient engagement experience are addressed by eHealth interventions?

## Methods

### Search strategy

A literature review was conducted in 2014 on four databases (PsychInfo, Scopus, Web of Knowledge, and PubMed), considering the last 10 years. This systematic review was conducted according to the PRISMA guidelines (a protocol used to perform systematic reviews) (Liberati et al., 2009).

The choice of databases was determined by the fact that the field of interest for this review is deeply multidisciplinary. The search was performed using thesaurus and free text terms, combining the words appropriately. The foci of the search were “e-health” and “engagement” and “activation.” We decided to consider both the term “engagement” and “activation” as they are used as synonymous in the literature and both refer to a complex vision of patient engagement. A list of keywords was created around the domain of “eHealth” based on the current literature about this topic; precisely, as other authors of recent systematic reviews on the eHealth topic have done (Eland-de Kok et al., 2011; Linn et al., 2011), we focused on internet-related technologies and generated a search string which entails the most widespread terms associated with the field. So, a search string was constructed as follows: engagement OR activation AND [“e-Health” OR “eHealth” OR “telemedicine” OR “tele-health” OR “telecare” OR “health information technology” OR “health information systems“ OR “interactive health communication”].

### Selection of articles

Two-step screening of all publications retrieved by the first scan was conducted in parallel by two authors (SB, ST) to determine eligibility for further review. Authors resolved disagreements through consensus. Authors first screened titles and abstracts of each contribution. Only peer reviewed research papers published in English were considered. Then, we applied the following eligibility criteria as the first step of screening:

1. The eHealth actions described must have been performed for the engagement of patients (technologies applied to engage other health stakeholders such as medical staff, hospital managers, or others were excluded);
2. The intervention had to feature at least one group of participants (single cases excluded); both between and within groups designs were considered;
3. The intervention had to assess one or more variables connected to patient engagement.

The subsequent full text analysis of the retrieved publications, performed independently by three authors (SB, ST, CL), allowed us to exclude further publications due to the following reasons: (1) the interventions used not well-specified technologies, or the technologies used were not clearly internet-based (i.e., telephone); (2) the terms “patient engagement,” or “patient activation” were actually present in the paper, but there were not references to the construct (i.e., unspecific use of the terms) and there were not appropriate measures to assess it; (3) despite exploring topics of interest for the health debate, the research didn't involve real patients (i.e., interventions with simulated patients) or was not focused on clinical populations (i.e., health promotion interventions).

All publications that met these eligibility criteria (first and second screening steps) were compiled to obtain the final sample. The authors used the same coding scheme to analyze the retrieved contributions. A discussion between the research team resolved the few minor differences that emerged in the coding process.

Each contribution was coded according to the following thematic categories: name of the authors and year of publication, research design, sample, eHealth tools, patient engagement related outcomes (Table 1). Also a Table 2 is available in the Discussion section, where the papers are again categorized highlighting the measures associated with patient engagement and, considering the main theoretical models in the field, the domain of patient engagement addressed (behavioral, cognitive, emotional).

**Table 1:** **Summary of intervention studies with patient engagement outcomes**.

**Paper**	**Design**	**Sample**	**Intervention (e Health tools)**	**Patient engagement related outcomes**
Aberger et al., 2014	One group of patients reporting self-monitored blood pressure	66 post renal transplant patients	A tele-health system that incorporates electronic blood pressure (BP) self-monitoring by the patients, uploading to a patient portal and a Web-based dashboard that enables clinical pharmacist collaborative care in a renal transplant clinic	−75% of patients monitored themselves at least once, and 69% achieved the minimum of six readings and obtained a BP average
Agarwal et al., 2013	One group of questionnaire respondents (model estimated with moderated multiple regression)	283 adult chronic patients	PHRs (health information management tools to store, retrieve, and manage personal health information and stimulate health action)	- significant relationship between satisfaction with health care provider and intentions to use the tool - significant positive interaction between the perceived value of the tool and patient activation in their effects on intentions to use
Meglic et al., 2010	Pilot study comparing two groups of patients receiving treatment as usual (physician visits and antidepressant treatment) and treatment as usual with eHealth intervention	46 patients with depressive disorders	Web-based information and communication technology system, to support collaborative care management and active patient engagement, and online and phone-based care management performed by trained psychologists	- higher medication adherence - reduced depressive symptoms - higher perception of care quality - improved access to care - improved access to information
Quinn et al., 2011	cluster-randomized clinical trial with four groups (control–usual care vs. coach-only vs. coach PCP portal vs. coach PCP portal with decision support)	163 adult diabetes patients	- mobile application coaching - patient/provider web portals	No appreciable differences between groups for patient-reported diabetes distress and depression
Robertson et al., 2006	One group, repeated measures	144 depressed patients	RecoveryRoad that is a eHealth system designed to augment the routine clinical treatment of depression	- high adherence to the system - average depression severity declined from severe to mild - both clinicians and patients were generally satisfied with the programme and reported that it improved clinician-patient relationships
Saberi et al., 2013	Pilot study with qualitative methods	14 HIV-positive young patients	A tele-health medication counseling session	- Qualitative findings: the eHealth tool was effective in improving the quality of patient-provider dialog
Schrader et al., 2014	A pilot study testing the feasibility of the program (one small group of patients)	8 recently hospitalized patients from rural areas	An online-management program for both patients and health care workers, accessible by either Web-enabled mobile phone or Internet, enabling patient-clinician communication	- Qualitative findings: patients' low information technologies literacy, interaction problems related to the illness conditions and technical limitations (for example: drop-out of rural Internet connections) constitute barriers to the technology enhancing patient engagement
Sharry et al., 2013	One group, repeated measures	80 university students with depression symptoms	An online, therapist-supported, CBT-based program for depression	- high level of engagement compared to a previous study - significant decreases in depression symptoms after the intervention
Solomon et al., 2012	Randomized control trial with two groups. (The participants in the Intervention Group had access to MyHealth Online, a patient portal featuring interactive health applications accessible via the Internet. Control group had access to a health education website featuring various topics). Parametric statistical models (*t*-test, analysis of variance, analysis of covariance) were applied to draw inferences	201 chronic adult patients (diagnosed with asthma, hypertension, or diabetes)	Web-based intervention on the patient activation levels for patients with chronic health conditions, measured as attitudes toward knowledge, skills, and confidence in self-managing health	- improvements (positive and significant effect) in patients' knowledge, skills, and self-efficacy to self-manage their health in the intervention group
Tang et al., 2013	Randomized controlled trial—intervention (INT) vs. usual care (UC)	415 patients with type 2 diabetes	The intervention included: (1) wirelessly uploaded home glucometer readings with graphical feedback; (2) comprehensive patient-specific diabetes summary status report; (3) nutrition and exercise logs; (4) insulin record; (5) online messaging with the patient's health team; (6) nurse care manager and dietitian providing advice and medication management; and (7) personalized text and video educational ‘nuggets’ dispensed electronically by the care team	- the intervention group had significantly better control of their LDL cholesterol at 12 months - the intervention group had significantly lower treatment-distress scores compared with those in the usual group - the intervention group also had greater overall treatment satisfaction and willingness to recommend treatment to others at 12 months. - enhanced active participation in measurement and communication with the health providers
Vest and Miller, 2011	One group (model estimated with ordinary least square regression)	3278 hospitals (patients' self-reports about satisfaction were assessed)	Implemented HIE (information technology for inter-organizational sharing of patient information)	- hospitals' level of HIE not associated with the percentage of patients reporting doctors communicated well. - implemented HIE associated with the percentage of patients who reported nurses always communicated well and who would definitely recommend the hospital.

**Table 2:** **Domains of patient engagement addressed in the retrieved studies**.

**Paper**	**Assessment (variables/aspects associated with patient engagement)**	**Domains of PE addressed**
Aberger et al., 2014	- Percentage of patients actively adhering to the system	Behavioral (self-monitoring)
Agarwal et al., 2013	- a validated 3-item measure for future use intentions	Emotional (care satisfaction, self confidence), behavioral (activation and self management skills) and cognitive (self efficacy, motivation to care, health knowledge)
	- the patient activation scale (PAM) from Hibbard et al.	
Meglic et al., 2010	- Questionnaire to assess medication adherence combining 3 previously reported measures: (1) regularity of administration over the defined medication period, (2) taking the medication at the same time of the day, and (3) regular use of correct dosage - Beck Depression Inventory-II to assess reduction in depression severity - open-ended questions on patient perception of care quality, access to care, and access to information	Emotional (depression, satisfaction toward care) and behavioral (adherence to medication, access to care) and cognitive (access to information)
Quinn et al., 2011	- The Patient Health Questionnaire-9 (PHQ) to assess depressive symptoms - the 17-item Diabetes Distress Scale	Emotional (depression, diabetes distress)
Robertson et al., 2006	- Adherence to the system; - self-reported medication adherence; - depression severity (Depression Severity Scale); - patients' ratings of satisfaction with the system	Emotional (depression, satisfaction) and behavioral (adherence to the system)
Saberi et al., 2013	Qualitative interviews to patients who had a high level of self-reported adherence to the system	Considering the themes emerging from the interviews; the eHealth intervention “leads to more disclosure” (behavioral), “improves health education” (cognitive), “increases patient comfort” (emotional)
Schrader et al., 2014	Qualitative interviews about obstacles/opportunity for the technology implementation	Behavioral, cognitive (i.e.,: the interviews highlight mostly important conditions for the technology correctly working and being used effectively for patient engagement)
Sharry et al., 2013	- behavioral variables of engagement understood as tool usage (number of sessions completed, mean time spent on the program, etc…)	Behavioral, emotional (depression)
Solomon et al., 2012	Patient Activation Measure (Hibbard et al., 2004) to assess patients about their attitudes toward knowledge, skills, and confidence in self-managing health	Emotional (self-confidence), behavioral (change in health behavior; adherence to prescribed medication regimens, regularly testing glucose levels, and monitoring blood pressure) and cognitive (health literacy)
Tang et al., 2013	- Diabetes Knowledge Test—a 14-item assessment of knowledge about diet, glycemic control, glucose testing, complications and insulin-use - Problem Areas in Diabetes—measures diabetes-related - stress in response to 20 common situations - Patient Health Questionnaire (PHQ-9)—depression screening tool - Diabetes Treatment Satisfaction Questionnaire (DTSQ) was used for the baseline assessment of total diabetes treatment satisfaction, treatment satisfaction in specific areas, and perceived frequencies of hypo- and hyper-glycemia - CAHPS assessed patient experience in access to care, clinician communication, shared decision making, and cost of care	Behavioral (self-monitoring), emotional (treatment satisfaction, emotional distress, patient-physician relation)
Vest and Miller, 2011	- Percentage of patients who reported satisfaction about communication with doctors and nurses - Percentage of patients who would definitely recommend the hospital and/or gave the hospital a high global rating	Emotional (satisfaction about communication and experience in the hospital)

## Results

### Main findings

**Figure 2:**
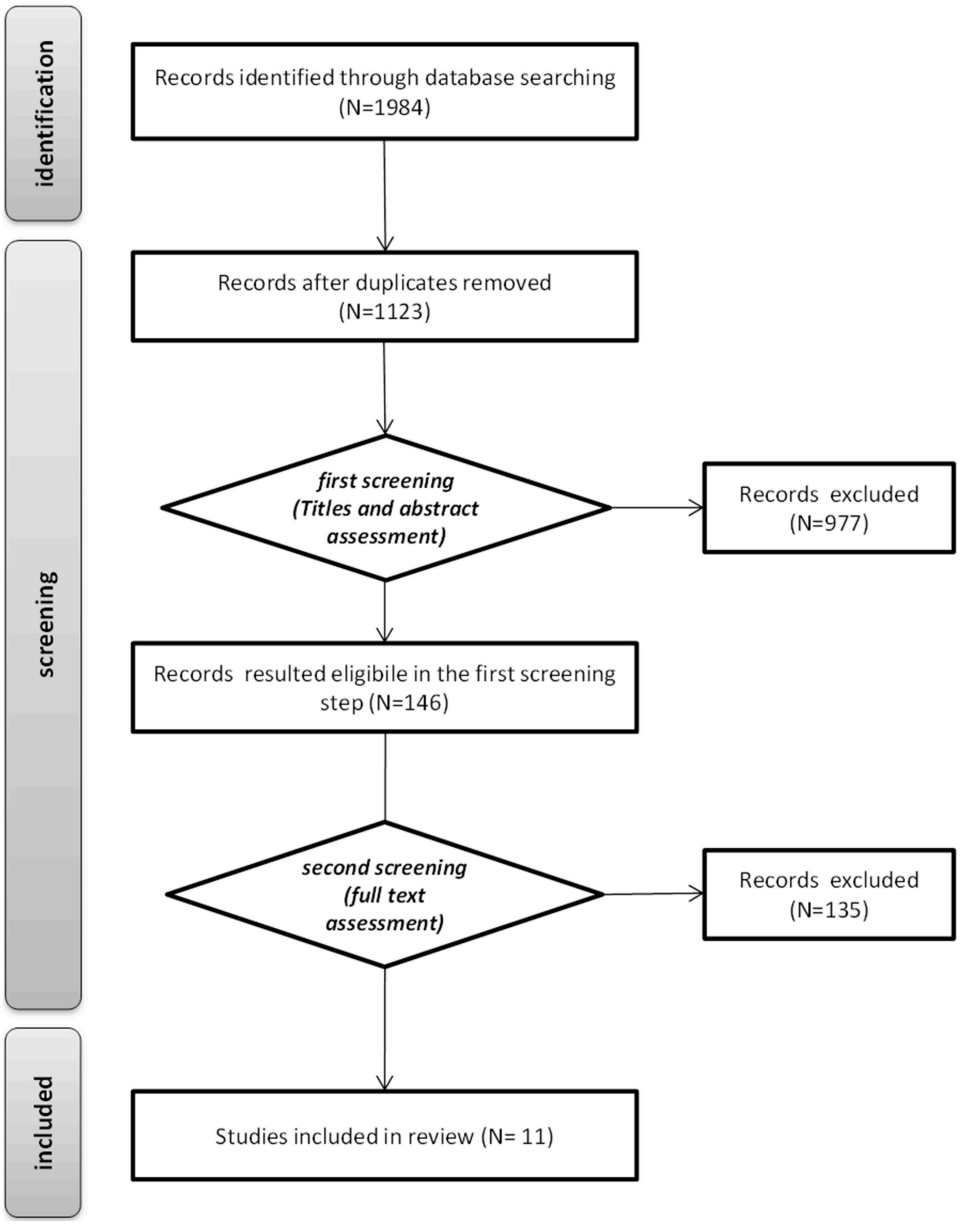
**Systematic review flow**.

The flowchart depicting the results of the literature selection process is shown in Figure 2. Our database queries resulted in 1984 sources. After removing duplicates, 1123 studies were left for more detailed examination. Titles and abstracts screening led to identify 146 articles met our first screening step inclusion criteria. Assessment of the remaining papers' full texts resulted in the second screening step basing on further exclusion criteria. In the end, 11 publications were identified as suitable to the inclusion criteria.

The features of the 11 papers that were included in the final sample of this review are discussed in detail.

### Key features of the selected studies

#### What patient engagement outcomes are discussed in eHealth interventions?

We here describe the retrieved papers, highlighting their results as regarding patient engagement-related outcomes. Table 1 shows an overview of the 11 papers included in our synthesis. It is relevant to note how patient engagement is the primary objective for some of the included studies, while for others it is treated as a secondary objective. For example, Agarwal et al. (2013) investigated how individual and technological/organizational factors affected the patients' attitude to use eHealth tools related to Personal Health Records (PHRs). In this study, patient engagement is assessed by using the Patient Activation Measure (PAM) by Hibbard et al. (2004), that is a measure of patients' ability to effectively manage their health and healthcare. Agarwal and colleagues' sample was composed by 283 patients, early adopters of the PHRs tools, affected by different chronic conditions. Among the different results, the authors identified a positive interaction between patient activation and the perceived value of the tool in their effect on intentions to use it. This means that, as regarding the intention to use a eHealth tool that is perceived as a useful resource to manage their own care, the more engaged patients obtained the highest results.

The PAM by Hibbard was also used by Solomon et al. (2012) as their primary outcome measurement tool. They were interested in evaluating the use of eHealth tools with patients with chronic diseases. The activation of patients seem to be particularly important in the context of these patients' care, because they have to daily confront with the self-management of the pathological illness condition. In this sense, self-management programs are designed to teach skills to promote self-care behaviors in patients, and also to foster self-confidence in their perceived abilities to manage their own health condition. In this study 201 chronic adult patients were divided in two groups. The ones in the experimental group had access to MyHealth online, a web portal featuring interactive health applications accessible via the internet, while the ones in the control group had access to a health education website. The PAM scores revealed a significant difference between the two groups, with the experimental group showing an increasing of patient activation after the intervention.

Also Tang et al. (2013) considered chronic pathological conditions, focusing on patients with type 2 diabetes. The diabetic patients (*N* = 379) were divided in two groups, experimental (using EMPOWER-D, a complex eHealth platform of tools to daily manage the disease: see the research in Table 1 for details) and control (receiving standard care). The patients in the experimental group showed much control of their own LDL cholesterol than the control group. Moreover, they reported much satisfaction, lower treatment distress and participated more actively in measurement and communication with health providers. Using a specific research perspective, Vest and Miller (2011) evaluated 3278 hospitals confronting their level of implemented HIE (Health Information Exchange, i.e., the inter-organizational sharing of patient information) with the levels of patient-provider communication and patient satisfaction. Although in this case there is not a technology which is directly used by patients, the use of information technologies at an organizational level is expected to affect emotional engagement of patients (Hripcsak et al., 2009). Through an ordinary least square regression model, they found that level of implemented HIE did not predict percentage of patients reporting doctors communicated well, but did predict percentage of patients who reported nurses communicated well and who would recommend the hospital. In this case, the implementation of a eHealth technology at an organizational level appeared associated with patient's satisfaction of care experience and care relationships. Also Aberger et al. (2014), who tested a tele-health system featuring blood pressure self-monitoring by 66 post-renal transplant patients, reported percentages about patients' adherence to the system as a measure of patient engagement; 75% of patients monitored themselves at least once, while 69% achieved the minimum of six readings and obtained a BP average. Quinn et al. (2011) conducted a research with diabetic patients to test whether adding mobile application coaching and patient/provider web portals to community primary care compared with standard diabetes management would reduce glycated hemoglobin levels in patients with type 2 diabetes. They found that the combination of behavioral mobile coaching with blood glucose data, lifestyle behaviors, and patient self-management data individually analyzed and presented with evidence-based guidelines to providers substantially reduced glycated hemoglobin levels over 1 year. In this research, eHealth technologies (i.e., mobile application and web portals) have been shown to directly-impact on depressive symptom reduction and on improved patient's self-management. Another notable case is the research by Sharry et al. (2013) on depressive symptoms. The authors discuss the benefits of implementing an online CBT based program for treating depressive disorders. Results showed high level of patient engagement with the online interactive therapist-supported program with a significant reduction in depressive symptoms following program completion. Moreover, the system described in the paper also illustrates how eHealth technologies can enhance patient engagement by using a range of design strategies intended to improve the user experience. Robertson et al. (2006) described their findings from the implementation of a comprehensive e-health system, named RecoveryRoad, which was designed to augment the routine clinical treatment of depression and patients' engagement in their care. Results showed that adherence to the system was high and self-reported medication adherence was over 90%. Moreover, patients reported an average depression severity declining from severe to mild thus showing that their engagement with the system had an impact on clinical outcomes. Moreover, the majority of patients surveyed were satisfied with the system itself and reported that it increased their knowledge of depression, and enhanced the relationship with their clinicians. Valuable insights also come from the two qualitative studies included in the present review (Saberi et al., 2013; Schrader et al., 2014); the first involved 8 recently hospitalized patients from rural areas, testing the feasibility of an online management program which enabled communication and engagement between patients and healthcare professionals. The results highlight some possible barriers limiting the effectiveness of the technology-based intervention, such as low information technologies literacy, interaction problems related to the illness and technical problems. Conversely, the study by Saberi et al. (2013) focused on the positive outcomes of a tele-health medication counseling session for 14 HIV-positive young patients. They provide rich information about the positive response from the patients. The technology-mediated session was considered less intimidating than the in-person visit and adequate for self-disclosure, and also useful in promoting patients' health education and engagement in treatments.

#### How patient engagement outcomes are assessed in eHealth interventions (i.e., instruments and methods)? what dimensions (cognitive, behavioral, emotional) considered by the current frameworks of patient engagement are actually addressed?

The analysis of the retrieved study showed that quite different measures were used to assess patient engagement (see Table 2). This appears consistent with the literature on the topic, which highlights a multiplicity of dimensions affecting patient engagement.

In Table 2, the reviewed articles are categorized basing on the patient engagements' dimensions measured during the intervention. Now we in-depth discuss the dimensions as they were conceived in the reviewed studies, and the variables associated to them that have been actually measured.

#### Behavioral and cognitive dimensions

The most systematically assessed dimensions of patient engagement are those involving cognitive and behavioral activation. According to this perspective, engaged patients enact more adaptive behaviors that are related to their active participation in their own care and show improvement in their aware access, use and sharing of healthcare information. This ends in the patient's acquiring new skills to effectively manage their illness experience.

Classically, in this research field behaviors are often observed through the monitoring of interactions with the eHealth tools. For example, to obtain a marker of their health engagement, the technological system counts the number of accesses by the patients, or collects the mean time spent on a page/function of the web resource. In the reviewed papers, similar behavioral variables were considered (Robertson et al., 2006; Sharry et al., 2013). Another type of patient's behavior used to assess engagement is adherence to medical prescriptions rather than to the eHealth tools. For example, Meglic et al. (2010) administered a questionnaire to assess regularity of medication administration, taking the medication at the same time of the day and regular use of the correct dosage. Also other authors (Solomon et al., 2012; Tang et al., 2013) assessed similar variables. Aspects related to behavior are also those connected with patients' abilities in the use of the technology; the state of illness may feature physiological/cognitive constrains that generate difficulties in using an eHealth platform, this way limiting its value for patient engagement (Schrader et al., 2014). Indeed, behavioral variables are sometimes overlapped with cognitive ones. This also happens when participants are asked about their habits and/or attitudes. In this sense, they do not only report if they performed a certain behavior or not (and how much they did), but also they express a cognitive structure related to a habitual way to represent behavior and actions. For this reason, the PAM (Hibbard et al., 2004) actually could be considered both a measure of behavioral and cognitive variables. However, cognitive variables could be associated with other aspects of patients' experience. There is generally consistency between the papers considered in this review, for what regards cognitive variables. Two of the considered studies (Solomon et al., 2012; Agarwal et al., 2013) used the PAM. Some research (Meglic et al., 2010; Tang et al., 2013) assessed patients' access to medical information and their knowledge about the pathological condition and the therapy's practices.

#### Emotional dimension

Another dimension of patient engagement addressed by the considered research is the emotional one. Authors conceive the emotional dimension as twofold: on the one hand, they assess clinical variables connected to the emotional state of the patient (i.e., depression, distress, or anxiety). On the other hand, they directly evaluate the patient's emotions related to their care experience. As regarding research assessing emotional variables, Robertson et al. (2006) found significant decrease of the depressive symptoms after the intervention. Similar results were found by Sharry et al. (2013) and Meglic et al. (2010) when assessing their eHealth interventions, while Quinn et al. (2011) found no significant differences in depressive symptoms related to their mobile application/patient-provider web portals intervention directed to diabetic patients. They also didn't find significant differences as regarding diabetes related distress. Differently, Tang et al. (2013) identified the emotional distress after the eHealth intervention for diabetic patients, confronting the experimental group with the control one. The other studies considered care satisfaction measures, which also resulted often positively affected by eHealth interventions (Robertson et al., 2006; Meglic et al., 2010; Vest and Miller, 2011; Tang et al., 2013). The contribution that provided the more fine-grained information about emotional response by the patients is the one by Saberi et al. (2013): the eHealth intervention was found positive by the patients, who highlighted its feasibility in promoting their own self-disclosure about treatment difficulties and in improving their comfort in the context of the communication with the physician.

## Discussion

Patient engagement is a primary goal for worldwide healthcare interventions (Hardyman et al., 2015). Moreover, eHealth technologies have often been found as an effective resource to foster the active role of patients in their healthcare (Gruman et al., 2010; Wasson et al., 2012; Bornkessel et al., 2014) beyond improving health outcomes. For this reason, eHealth interventions recently started to also assess variables associated with patient engagement. Despite this, we found that these variables are often heterogeneous; not only for what concerning the assessment meaures/instruments, but also they often appear related to different dimensions of patient engagement. In this regard, Table 3 shows the complete list of the patient engagement related variables assessed by the reviewed studies. Independently by the theorethical stance we based on conducting this review, which categorizes the variables as behavioral, cognitive and emotional, it is clear how very different and still not-integrated approaches exist in the literature. Moreover, the examined studies lack in a systematic assessment of the level of patient engagement/activation pre and post intervention. Future studies may consider—according to the most established theoretical models—to examine not only the impact of the eHealth on patient engagement, but also to assess who uses eHealth (the more or less engaged), and who eHealth helps the most (the more or less engaged).

**Table 3:** **The distribution of the patient engagement variables assessed by the reviewed studies**.

**Paper**	**Access to eHealth system**	**Adherence to treatment/medication**	**Abilities in the use of the eHealth system**	**Health management habits**	**Knowledge of the disease**	**Depressive symptoms/Emotional distress**	**Satisfaction/Positive emotions**
Aberger et al., 2014	X	X	–	–	–	–	–
Agarwal et al., 2013	–	–	–	X	–	–	X
Meglic et al., 2010	–	X	–	–	X	X	X
Quinn et al., 2011	–	–	–	–	–	X	–
Robertson et al., 2006	X	–	–	–	–	X	X
Saberi et al., 2013	–	–	–	–	–	–	X
Schrader et al., 2014	–	–	X	–	–	–	–
Sharry et al., 2013	X	–	–	–	–	X	–
Solomon et al., 2012	–	X	–	X	–	–	–
Tang et al., 2013	–	X	–	–	X	X	X
Vest and Miller, 2011	–	–	–	–	–	–	X

On these basis, we decided to provide a review which is two-fold in its results. On the one hand, we described the main patient engagement outcomes of eHealth interventions; they appear to confirm how internet technologies in healthcare are able to give patients a starring role in their own healthcare. On the other hand, we highlighted the *methods* that are currently associated to the patient engagement assessment in eHealth interventions. Probably denoting the actual limitations in the broader literature on the topic, these methods have been often found simplified and/or partial in their explanatory power about patient engagement (Graffigna et al., 2015a,b). A number of methods were focused on the individuals' behavioral activation: those studies assessed patient's adherence to medical prescriptions, which was registered as an indicator of their actual engagement. Despite being a fundamental variable, this type of behavioral marker is not totally descriptive of the whole patient's subjective care experience. Other methods also considered variables related to the *cognitive* dimension of engagement, such as the patient looking for information and elaborating it in order to better understand and manage his/her own disease condition. Last but not least, the *emotional* dimension has been sometimes considered only in the form of clinical variables (e.g., depressive symptoms) or the level of patient satisfaction toward the received care—otherwise only in the more recent literature. Hovewer, Graffigna et al. (2013a, 2015b) suggest that the emotional dimension also involves the patients' acceptance of the disease; ways of affective adjustment to the illness course; the level of patients' quality of life (Barello and Graffigna, 2014; Graffigna et al., 2015b); the quality of patient-doctor relationship (Barello and Graffigna, 2014, 2015); meaning making processing as affected by the affective quality of the care experience.

While instruments such as the PAM by Hibbard et al. (2004) offer an adequate resource to monitor behavioral and cognitive aspects of patient activation, the emotional dimention of the engagement experience still needs further consideration and requires specific assessment tools (Graffigna et al., 2015a). Precisely, the patients' illness experience, and what he *feels* when taking part in a healthcare intervention, could be monitored thanks to the use of technologies. According to some authors (Hibbard and Mahoney, 2010; Carman et al., 2013), patients who are low activated are more likely to be weighted down by negative affect which can lead to a cycle of negative self-perception. The negative affect can reduce the likelihood of attending to and using new information, and reduce openness to change.

All of these considerations point to the need to attend to emotional elaboration of the health experience as part of engagement strategies and eHealth can be part of this process. Indeed, new technologies are able to register and monitor behavioral, physiological and emotional variables during daily life, also providing immediate feedback to the patients (Riva, 2009; Gaggioli et al., 2011; Serino et al., 2014; Graffigna et al., 2014a). This could be done also with self-report measures, asking the patient to report on different emotions. In this sense, eHealth can be surely confirmed as an important driver for patient engagement in healthcare. However, further research is needed to develop complete and shareable methodologies for the accurate monitoring and analysis of patient engagement. Moreover, future research may be devoted to deepen the role of new technologies in promoting patient engagement. Precisely, recent advances in the eHealth field focus on specific technologies which are used to deliver specific types of interventions (for example, mobile communications in order to foster ubiquity and personal customization: mHealth (Istepanian et al., 2006; Bashshur et al., 2011); or health-related information delivered through gaming contexts; gHealth (Ferguson, 2012); and so on). These specific types of eHealth technologies may be object of interest for future reviews, also regarding their possible specific effects on the patients' experience of health engagement.

## Conclusion

To sum up, the eHealth interventions we reviewed were mainly devoted to foster only one or two experiential dimensions of patient engagement (i.e., alternatively cognitive, emotional or behavioral experiential dimensions related to the healthcare management), thus not considering the complexity of this experience, due to the interrelation among different psychological domains of patient subjectivity. This also led to a great heterogeneity of technological tools, practices and of achieved results. Finally, although eHealth technologies supposedly hold patient autonomy and proactivity in self-care as the ultimate goal of eHealth, our results showed a still passivizing logic—although implicit—in the implementation of eHealth interventions due to the patients' low engagement in the development/design of the care process. Despite that, a possible limit of the present review is the still existing paucity of studies investigating the relationship between eHealth and patient engagement—assuming the wider conceptualization of this term—, thus resulting in a limited number of publications considered by the present review. The reason of that surely relies on the infancy of the literature on this topic (Menichetti et al., 2014). Moreover, we deliberately choose to focus on research whose authors explicitely reffered to the engagement/activation construts. Future reviews may consider specifically other related constructs such as patient adherence/compliance but they should taking into account that such a choice implies the risk of considering only partial aspects (i.e., the behavioral ones) of the patient illness experience. However, the retrieved studies show a promising role of eHealth for this purpose. In this sense, the present review constitutes a first step in order to develop more precise guidelines for designing and implementing eHealth interventions for patient engagement.

Although scholars agree on the importance of tailoring eHealth interventions on the basis of the deep understanding of the patient experience, this goal was not often achieved in the analyzed papers. This review underlines the need for a more holistic view of patient needs and priorities to directly engage them in the management of their care and to better shape eHealth strategies. This constitutes the main actual challenge of implementing eHealth interventions that are truly able to foster patient engagement. Failing to achieve synergy among the dimensions featuring the patients' care experience (i.e., cognitive, behavioral, and emotional) and lacking in globally considering their role in enhancing their engagement toward the care process, may result in limiting the benefit from the provided interventions (Graffigna et al., 2013a).

According to this requirement, we contend that the quality of patient experience should become the guiding principle in the design and development of eHealth interventions (Riva et al., 2012; Graffigna et al., 2015b). This may be the key in better orienting eHealth interventions to foster patient engagement.

Reaching a more holistic and systematic understanding of the patient engagement process may help in tailoring e-health interventions to be tuned to patient needs and priorities in each domain and at each phase of their health management experience (Graffigna et al., 2015b). Future research could offer a framework to orient eHealth intervention aimed at fostering patient engagement grouped for each experiential domain. The present review provides an overview of the main goals of eHealth that practitioners and academic institutions could use when developing interventions to promote patient engagement in their care plans.

### Conflict of interest statement

The authors declare that the research was conducted in the absence of any commercial or financial relationships that could be construed as a potential conflict of interest. The handling Editor declared a shared affiliation, though no other collaboration, with the authors [Stefano Triberti, Guendalina Graffigna, Chiara Libreri, Giuseppe Riva] and states that the process nevertheless met the standards of a fair and objective review.
